# Changes in soil organic carbon components and microbial community following spent mushroom substrate application

**DOI:** 10.3389/fmicb.2024.1351921

**Published:** 2024-05-17

**Authors:** Guiting Yang, Yan Ma, Xiaochi Ma, Xuanqing Wang, Chao Lu, Wenyi Xu, Jia Luo, Dejie Guo

**Affiliations:** ^1^Institute of Agricultural Resources and Environment, Jiangsu Academy of Agricultural Sciences, Nanjing, China; ^2^Key Laboratory of Saline-Alkali Soil Improvement and Utilization (Coastal Saline-Alkali Lands), Ministry of Agriculture and Rural Affairs, Nanjing, China; ^3^National Agricultural Experiment Station for Agricultural Environment, Nanjing, China; ^4^Department of Geosciences and Natural Resource Management, University of Copenhagen, Copenhagen, Denmark

**Keywords:** spent mushroom substrate, SOC fraction, microbial community, function prediction, ^13^C NMR

## Abstract

While spent mushroom substrate (SMS) has shown promise in increasing soil organic carbon (SOC) and improving soil quality, research on the interplay between SOC components and microbial community following the application of diverse SMS types remains scant. A laboratory soil incubation experiment was conducted with application of two types of SMSs from cultivation of *Pleurotus eryngii* (PE) and *Agaricus bisporus* (AB), each at three application rates (3, 5.5, and 8%). Advanced techniques, including solid-state ^13^C nuclear magnetic resonance (NMR) and high-throughput sequencing, were employed to investigate on SOC fractions and chemical structure, microbial community composition and functionality. Compared to SMS-AB, SMS-PE application increased the relative abundances of carbohydrate carbon and O-alkyl C in SOC. In addition, SMS-PE application increased the relative abundance of the bacterial phylum Proteobacteria and those of the fungal phyla Basidiomycota and Ascomycota. The relative abundances of cellulose-degrading bacterial (e.g., *Flavisolibacter* and *Agromyces*) and fungal genera (e.g., *Myceliophthora*, *Thermomyces*, and *Conocybe*) were increased as well. The application of SMS-AB increased the aromaticity index of SOC, the relative abundance of aromatic C, and the contents of humic acid and heavy fraction organic carbon. In addition, SMS-AB application significantly increased the relative abundances of the bacterial phyla Firmicutes and Actinobacteria. Notably, the genera *Actinomadura*, *Ilumatobacter*, and *Bacillus*, which were positively correlated with humic acid, experienced an increase in relative abundance. Functional prediction revealed that SMS-PE application elevated carbohydrate metabolism and reduced the prevalence of fungal pathogens, particularly *Fusarium*. The application of high-rate SMS-AB (8%) enhanced bacterial amino acid metabolism and the relative abundances of plant pathogenic fungi. Our research provides strategies for utilizing SMS to enrich soil organic carbon and fortify soil health, facilitating the achievement of sustainable soil management.

## 1 Introduction

Soil organic carbon (SOC) regulates crucial soil processes and is vital for sustaining productivity in agricultural ecosystems (Kan et al., 2022). Organic fertilization enhances SOC accumulation, promoting sustainable crop production and soil health (Kalkhajeh et al., 2021; He et al., 2023). Soil microorganisms, the primary decomposers of SOC, play a pivotal role in soil nutrient cycling (Xu et al., 2021). Previous studies have highlighted that the incorporation of diverse organic materials can significantly reshape microbial community composition and fractionation of SOC (Li et al., 2022; Zheng et al., 2022; Shi et al., 2023), leading to alterations in both microbial community diversity and function. Thus, it is important to comprehensively investigate the effects of different organic materials on soil microbial communities so as to optimize organic fertilization strategies, improve soil quality, and boost agricultural productivity (Li et al., 2020).

Recent research has demonstrated a strong correlation between SOC fractions and microbial communities (Su et al., 2020; Zhang et al., 2021). Microbial biomass carbon (MBC), extractable organic carbon, and fulvic acid (FA), which are the active components of SOC, mainly influence the bacterial communities, whereas the recalcitrant components of SOC are more closely associated with the fungal communities (Xiang et al., 2017; Mayer et al., 2021). Long-term biochar application was found to increase recalcitrant C and significantly change the fungal community structure across all soil depths but have little effect on the bacteria (Ma et al., 2023). Organic matter incorporation alters not only the fractions but also the chemical structure of SOC. Solid-state ^13^C nuclear magnetic resonance (^13^C NMR) spectroscopy is extensively utilized to investigate the chemical structure of SOC (Mustafa et al., 2022). Chen et al. (2023) demonstrated that the long-term wheat straw addition boosts carbohydrate carbon in light fractions and coarse particulate organic matter, altering the soil bacterial community. Conversely, corn stover addition increases aromatic C in both coarse and fine particulate organic matter, significantly changing soil fungal communities (Chen et al., 2023). The O-alkyl C mainly originates from easily metabolizable organic matter, such as cellulose and hemicellulose (Preston, 1996; Christensen, 2020), while aromatic C represents recalcitrant C compounds that largely determine the structural stability of SOC. A 27-year field experiment showed that long-term manure application increased the aromatic fraction of SOC and the relative abundances of Acidobacteria and Actinobacteria (Li et al., 2018). Baumann et al. (2009) demonstrated that soil microbial communities were influenced by the content of aromatic C and O-alkyl C after the addition of wheat straw and eucalyptus residue, respectively. Although extensive research has underscored the impacts of the long-term incorporation of organic materials, especially crop residues, on SOC fractions and the microbial community during decomposition, some studies indicate that soil characteristics and microbial community composition respond swiftly to fertilization management changes. For instance, notable increases in microbial biomass and activity are often observed shortly after organic fertilization (Lazcano et al., 2013; Ren et al., 2021). The influence of short-term application of organic material on soil microbial community composition and function remains largely unknown, necessitating further efforts to bridge this knowledge gap.

Spent mushroom substrate (SMS) is a byproduct generated during mushroom production. Improper SMS handling can result in environmental issues such as air and water pollution (Leong et al., 2022a). Therefore, there is a growing interest in the effective recovery and utilization of SMS. Although numerous studies have affirmed the potential of SMS as an effective fertilizer (Pérez-Chávez et al., 2019), it is widely acknowledged that SMS composition varies significantly with the cultivated mushroom species (Suwannarach et al., 2022; Leong et al., 2022b). For instance, the substrate for cultivation of *Pleurotus eryngii* (PE) a primary decomposer, is mainly composed of wood fibers (e.g., sawdust), straw, and inorganic nutrients (Estrada et al., 2009; Zhai and Han, 2018). Conversely, *Agaricus bisporus* (AB), a secondary decomposer, requires a nitrogen and polysaccharide-rich substrate, typically a mixture of manure and cereal straw (Kerrigan et al., 2013). Composting SMS was considered the most efficient and economically viable method for its recycling. Compared to SMS-AB, SMS-PE contains more plant fiber residues, leading to increased O-alkyl C content in the soil, easily decomposed by soil microorganisms. In contrast, SMS-AB, which has undergone AB decomposition and composting, exhibits a higher degree of humification and may contain more stable organic carbon. However, research on the effects of SMSs with different C compositions on soil microbial communities and functions is limited.

In this study, we conducted a laboratory incubation experiment to investigate the response of soil microbial communities and functions to the short-term application of different SMSs. The specific objectives were as follows: (1) to investigate the effects of short-term application of two different types of SMS and their respective rates on soil nutrients, SOC fractions, SOC chemical structure, and microbial community composition; (2) to determine the correlations between soil bacterial and fungal community composition and the fractions and chemical structure of SOC; and (3) to predict the potential functions of soil bacteria and fungi in the different SMS application treatments using PICRUSt2 and FUNGuild analyses.

## 2 Materials and methods

### 2.1 Characterization of SMSs

Jiangsu Guannan Yuguan Modern Agricultural Technology Co., Ltd. (China) provided the spent substrates of PE and AB. After the mushrooms are harvested, the residual substrate was collected for static composting. The static pile was regularly turned using a turner to ensure adequate aeration, and the composting process was completed within 2 months. Supplementary Table S1 in our previous study (Yang F. et al., 2024; Yang G. et al., 2024) presents the relevant properties of the SMSs. The chemical composition of SMS-PE contained 11.2% alkyl C, 13.8% methoxyl C, 39.5% O-alkyl C, 10.6% di-O-alkyl C, 8.5% aromatic C, 5.6% phenolic C, and 10.8% carbonyl C, while SMS-AB comprised 21.9% alkyl C, 11.1% methoxyl C, 23.8% O-alkyl C, 6.8% di-O-alkyl C, 10.8% aromatic C, 8.8% phenolic C, and 16.8% carbonyl C (Yang F. et al., 2024; Yang G. et al., 2024).

### 2.2 Experimental design and sample collection

The soil was collected from the farmland (0-20 cm) of Duigougang Town (34°5′N, 119°12′E) in Guannan County, Jiangsu Province. The essential characteristics of the soil were as follows: pH of 8.01, SOC content of 20.21 g kg^−1^, total N (TN) content of 1.41 g kg^−1^, available phosphorus (AP) content of 19.47 mg kg^−1^, and available potassium content of 465 mg kg^−1^. After ground to <2 mm, the SMSs were mixed thoroughly with the fresh soil (equivalent to 1.5 kg dry weight), transferred into glass containers (15 cm in diameter, 15 cm in height), and placed in a dark incubator at 25°C for 115 days. Soil moisture was maintained at 50–60% of water-holding capacity during incubation. Three application rates for each SMS were established, which were 3% (low: PEL and ABL), 5.5% (medium: PEM and ABM), and 8% (high: PEH and ABH), respectively. Therefore, a total of seven treatments were set up, including a control (CK) without SMS. After cultivation, fresh soil samples were collected and divided into three parts: one was stored at −80°C for soil DNA extraction, another was kept at 4°C for soil MBC determination, and the remaining was air-dried for soil property analysis.

### 2.3 Analyses of soil basic properties and SOC fractions

Soil properties, including pH, mineral N (NH_4_^+^ and NO_3_^−^), AP, and TN, were analyzed using various methods detailed in the Supplementary Text S1. SOC was determined using the K_2_Cr_2_O_7_ oxidation method. SOC fractionation followed the differential solubility technique (Swift, 1996) adapted by Benites et al. (2003) in separating the FA and humic acid (HA). Soil samples were divided into light and heavy fractions using the density fractionation method (Janzen et al., 1992), and the total carbon content of each fraction (LFOC and HFOC) was determined. DOC was extracted using 0.5 M K_2_SO_4_ and measured using a total organic carbon analyzer (Elementar, Germany). Soil MBC was determined using the chloroform fumigation-extraction method.

### 2.4 ^13^C NMR spectra of SOC

Before measurement, the soil samples were repeatedly treated with a 10% (v/v) hydrofluoric acid solution to remove Fe^3+^ and Mn^2+^, enhancing the instrument’s signal-to-noise ratio (Schmidt et al., 2011; Berhe et al., 2012). Subsequently, the soil samples were rinsed four times with deionized water to remove any residual hydrofluoric acid. The soil sample was dried in an oven at 40°C and ground through a 60-mesh screen, preparing it for cross-polarization magic angle spinning ^13^C NMR spectrometry. The ^13^C NMR analysis was conducted using a Bruker AVANCE III 400 spectrometer operating at a ^13^C resonance frequency of 100.625 MHz. The samples were packed into a 4 mm diameter zirconium dioxide rotor and spun at a frequency of 5 kHz. The contact time was set to 10 milliseconds, and a recycle delay of 1 s was applied. Seven regions from the NMR analysis in each spectrum were obtained: 0–45 ppm (alkyl C), 45–60 ppm (methoxyl C), 60–93 ppm (O-alkyl C), 93–110 ppm (di-O-alkyl C), 110–142 ppm (aromatic C), 142–160 ppm (phenolic C), and 160–190 ppm (carbonyl C). Various indexes of soil organic matter stability were computed as follows: (a) carbohydrate carbons (CC) = C60–93 ppm/C0–190 ppm (Chen et al., 2023), and (b) aromaticity index (AI) = C110–160 ppm/C0–190 ppm (Kubar et al., 2021).

### 2.5 DNA extraction, PCR amplification, and Illumina Mi Seq sequencing

The total DNA of soil samples was extracted using the Fast DNA Spin Kits (MP Biomedicals, Santa Ana, CA, United States). DNA integrity and purity were assessed using 1% agarose gel electrophoresis. Additionally, DNA concentration and purity were accurately determined with a NanoDrop 1000 spectrophotometer (NanoDrop Technology, Wilmington, USA). For bacterial analysis, the primer set 515F and 806R were utilized to amplify the V3-V4 hypervariable regions of the bacterial 16S rDNA gene. For fungal analysis, the primer set ITS5-1737F and ITS2-2043F were employed to amplify the ITS region of fungi. The PCR products were retrieved using the E.Z.N.A.^®^ Gel Extraction Kit (Omega, USA), and the target DNA fragments were eluted with TE buffer. The library preparation was performed following the standard protocol of the NEBNext^®^ Ultra^™^ II DNA Library Prep Kit for Illumina^®^ (New England Biolabs, United States). The constructed amplicon library was subjected to sequencing on the Illumina Nova 6000 platform using PE250 sequencing (provided by Guangdong Magigene Biotechnology Co., Ltd., Guangzhou, China).

### 2.6 Bioinformatics and statistical analysis

Data normality and variance homogeneity were evaluated using the Shapiro–Wilk and Levene tests, respectively. The effects of SMS type and application rate on soil characteristics were analyzed by two-way ANOVA, followed by *post-hoc* analysis using Duncan’s test (significance level set at *p* < 0.05). Alpha diversity indices, Chao1 (richness) and Shannon (diversity), were calculated at the OTU level. Non-metric multidimensional scaling (NMDS) analysis, based on the Bray-Curtis distance algorithm, was performed to investigate the differences in microbial community composition among treatments. The linkages between soil properties and microbial composition (phylum level) were evaluated using the Mantel test, implemented in the “vegan” package of R software. PCA analysis was performed using Origin 2022b software to investigate the composition of bacteria and fungi phyla in relation to selected soil characteristics. To assess species composition differences among soil samples at the genus level, a heatmap was constructed using the “pheatmap” package in R software.

Additionally, Spearman correlation analysis was performed between the top 50 genera in relative abundance in bacteria and fungi and selected soil characteristics, utilizing the “pheatmap” function in R software. 16S rRNA sequencing data based on PICRUSt 2 analysis was used to obtain functional profiles. The FUNGuild predicted database^1^ was employed to predict the ecological functions of fungal communities, with confidence levels categorized as “probable” and “highly probable.”

## 3 Results and discussion

### 3.1 Soil properties

At the end of the 115-day experiment, both SMS type and application rate caused significant changes in soil properties (Supplementary Table S2). Both SMSs increased SOC, DOC, TN, AP, and TP contents and reduced soil pH, and a higher SMS application rate caused more pronounced changes. For a same SMS application rate, SOC, DOC, MBC, AP, and TP contents and pH were significantly higher in the SMS-PE treatment than in the SMS-AB treatment, whereas the opposite was true for NO_3_^−^-N content.

### 3.2 SOC fractions

A higher application rate of SMS-PE caused no significant change in HFOC content but significantly increased the contents of LFOC, FA-C, and HA-C (Supplementary Table S3). By contrast, a higher application rate of SMS-AB led to a higher HFOC content. For a same SMS application rate, soil LFOC content in the SMS-PE treatment was significantly higher than that in the SMS-AB treatment, whereas soil HA-C content in SMS-AB was significantly higher than that in SMS-PE.

### 3.3 Chemical structure of SOC fractions

**Figure 1:**
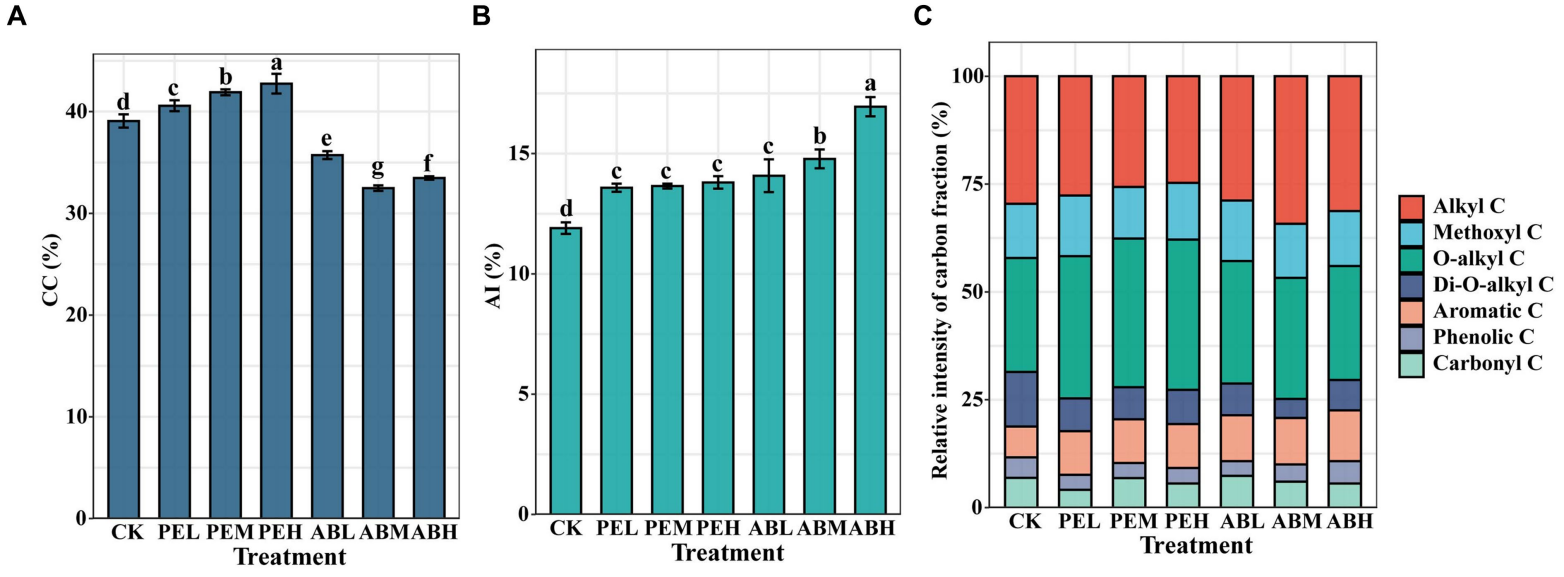
The proportion of CC **(A)**, AI **(B)**, and distribution of chemical shift ranges in total signal intensity (%) for ^13^C NMR **(C)** of soil organic C under different SMS application treatments. CC, carbohydrate carbons; AI, aromaticity index; SMS, spent mushroom substrate.

The components of SOC in all treatments were predominantly composed of alkyl C and O-alkyl C (Figure 1). The O-alkyl C was the most dominant SOC component in all SMS-PE treatments, and its proportion in SOC increased with increasing SMS-PE application rate. In comparison to CK, the proportions of soil CC significantly increased by 3.84, 6.96, and 8.77% in the PEL, PEM, and PEH, respectively (Figure 1A). However, regardless of the application rate, the proportion of soil CC in the SMS-AB treatment significantly decreased. SMS application significantly increased the AI of SOC in all treatments (Figure 1B). ABM and ABH exhibited significant increases in AI by 8.24 and 22.83% compared to PEM and PEH, respectively.

### 3.4 Soil microbial diversity and community structures

**Figure 2:**
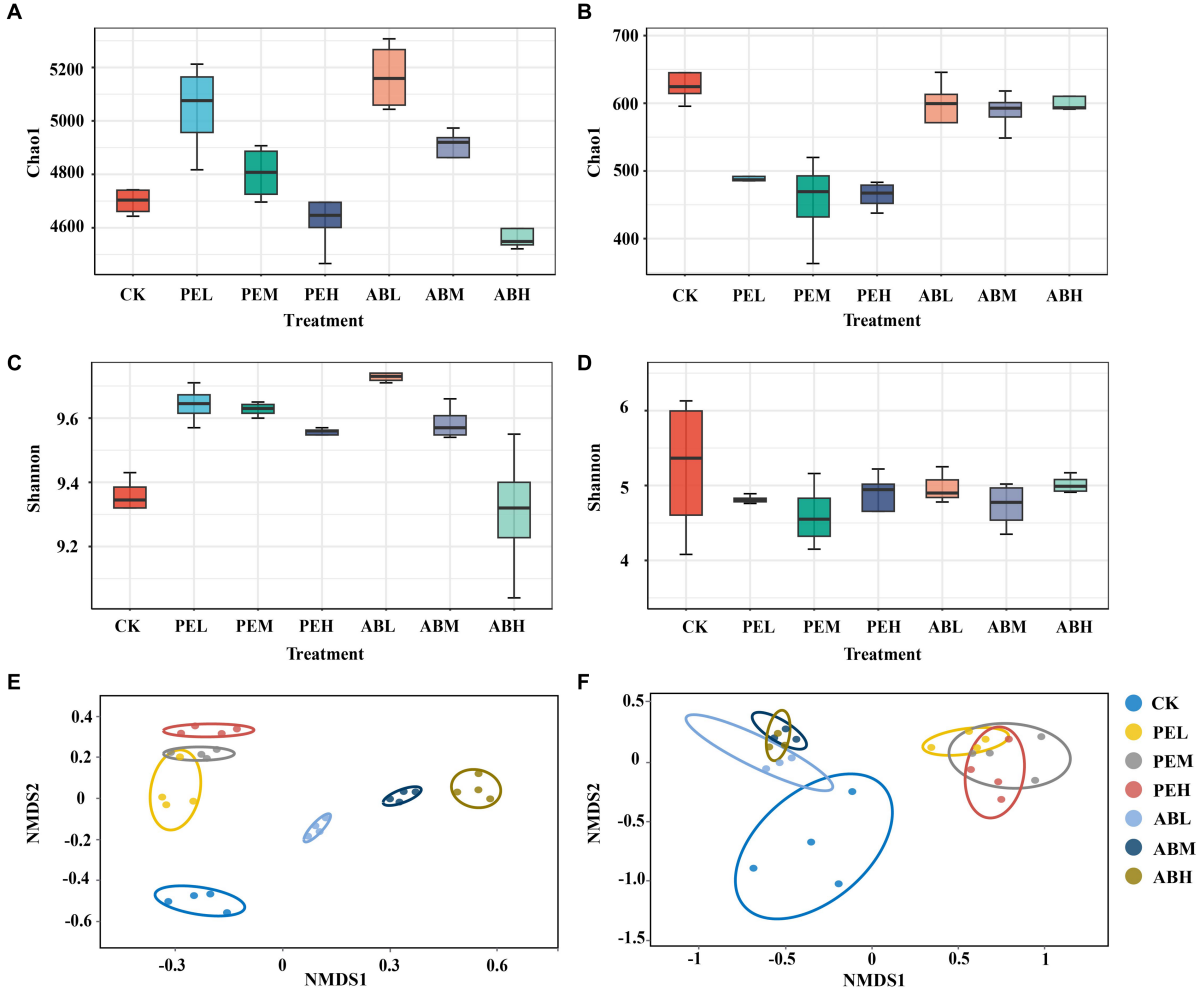
The Chao1 index, Shannon index, and non-metric multidimensional scaling (NMDS) plot based on Bray–Curtis dissimilarity of bacterial **(A,C,E)** and fungal **(B,D,F)** communities in soil under different treatments.

As the application rate of SMS increased, bacterial richness and diversity decreased (Figures 2A,C). In comparison to the CK, the application of SMS-AB did not have a significant effect on fungal richness. In contrast, SMS-PE application significantly reduced fungal richness (Figure 2B). The type and application rate of SMS had no significant effect on fungal diversity (Figure 2D). NMDS plots based on Bray-Curtis distance displayed seven distinct clusters of bacterial and fungal communities (Figures 2E,F). The PEL and PEM bacterial community clusters overlapped, whereas the other five clusters were clearly separated. The three SMS-PE (PEL, PEM, and PEH) fungal community clusters overlapped with each other, and the three SMS-AB (ABL, ABM, and ABH) fungal community clusters overlapped with each other. However, the two cluster groups were clearly separated from each other. These results indicate that both the type and application rate of SMS significantly affected the bacterial communities, whereas only the type of SMS significantly influenced the fungal communities.

**Figure 3:**
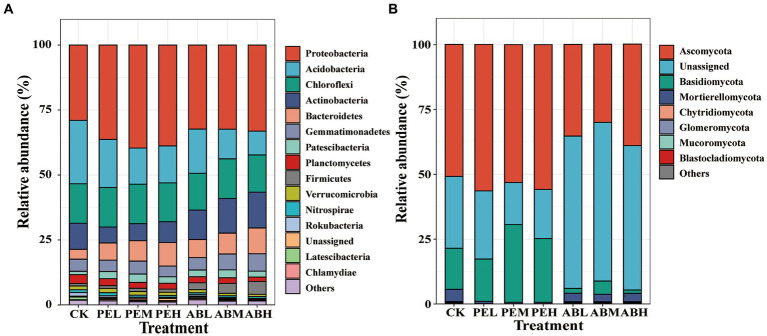
The average relative abundances of bacterial **(A)** and fungal taxa **(B)** at the phylum level under different treatments.

After 115 days of incubation, the predominant bacterial phyla were Proteobacteria, Acidobacteria, Chloroflexi, Actinobacteria, Bacteroidetes, Gemmatimonadetes, and Firmicutes in all treatments (Figure 3A). Compared to CK, SMS application decreased the relative abundance of Acidobacteria while increased the relative abundance of Bacteroidetes and Proteobacteria. Comparing the two SMSs, SMS-PE application increased the relative abundance of Proteobacteria, whereas SMS-AB increased those of Firmicutes and Actinobacteria. The predominant fungal phyla across treatments were Ascomycota and Basidiomycota (Figure 3B). The SMS-PE treatments increased Ascomycota and Basidiomycota relative abundances compared to CK, whereas the SMS-AB treatments decreased their relative abundances.

### 3.5 Correlations between soil properties and bacterial and fungal communities

**Figure 4:**
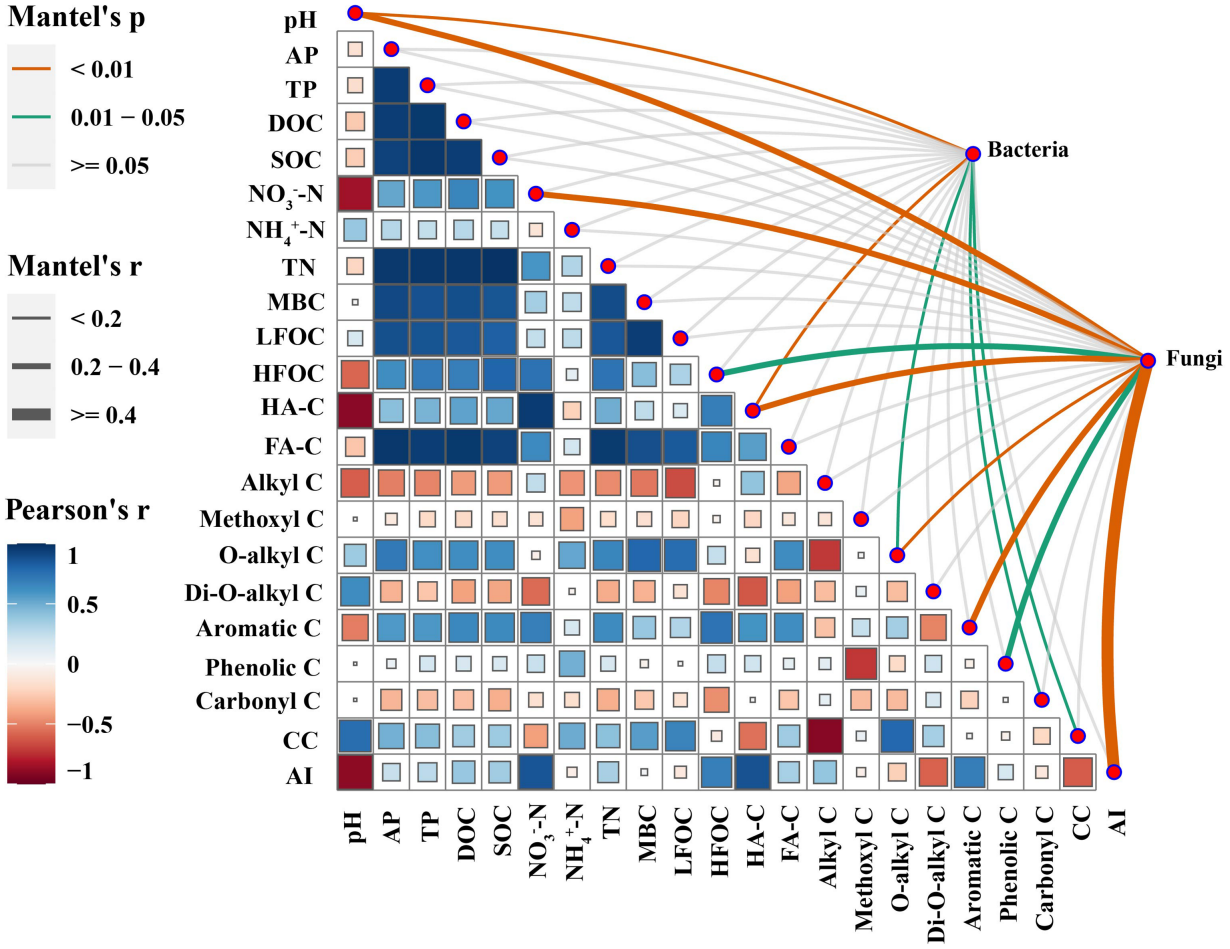
Correlations between soil microbial composition (at the phylum level) and soil properties identified by Mantel test.

The Mantel test indicated that the composition of bacterial phyla was significantly affected by pH, O-alkyl C, CC, HA-C, and Carbonyl C, while the composition of fungal phyla was significantly affected by pH, NO_3_^−^-N, HFOC, HA-C, O-alkyl C, Aromatic C, Phenolic C, and AI (Figure 4).

**Figure 5:**
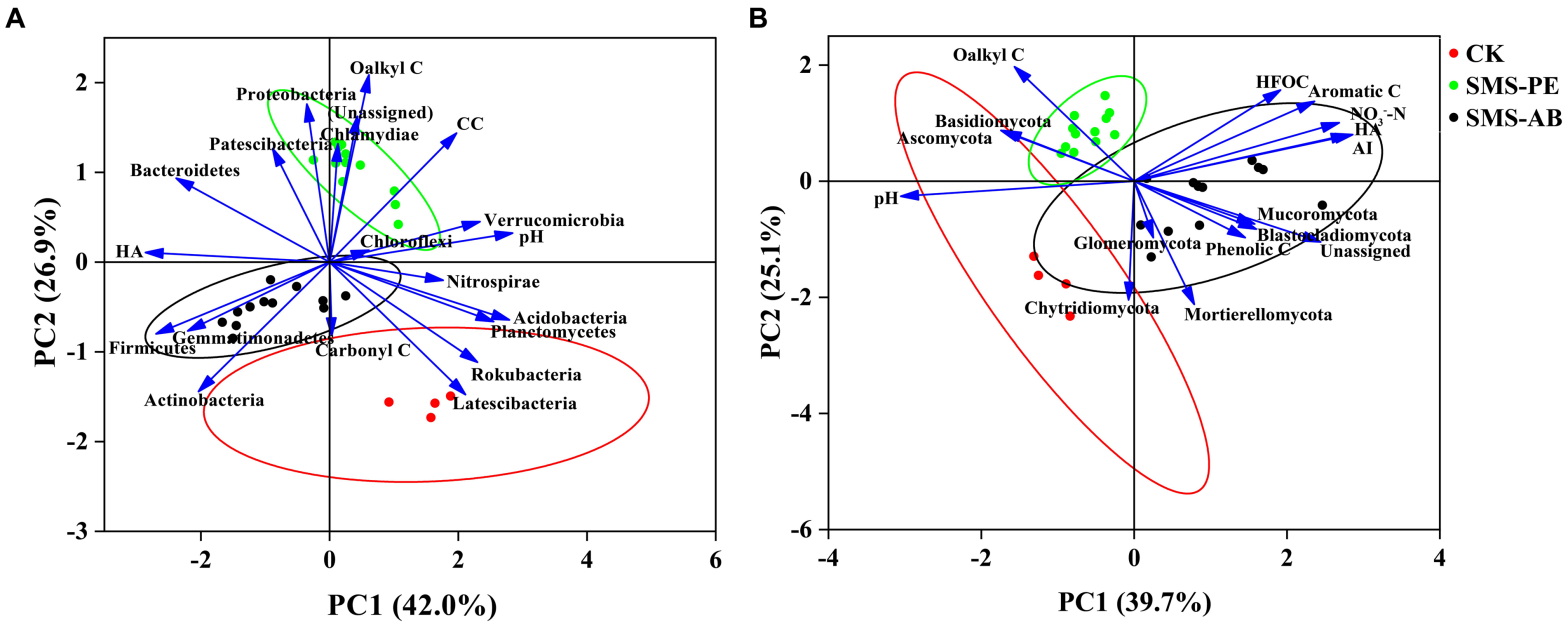
Principal component analysis (PCA) of major bacterial **(A)** and fungal phyla **(B)** and selected soil parameters.

PCA analysis showed that axes 1 and 2 accounted for 42.0 and 26.9% of the total variance in soil bacterial community composition, respectively (Figure 5A). There was significant difference in bacterial phylum-level community composition between the SMS-PE and SMS-AB treatments. The bacterial community compositions at the phylum level in the SMS-PE treatments were mainly associated with higher proportions of O-alkyl C and CC and a lower proportion of carbonyl C. The bacterial community compositions at the phylum level in the SMS-AB treatments were mainly related to a higher HA-C content, a lower pH, and lower proportions of O-alkyl C and CC (Figure 5A).

As shown in the PCA ordination plot, axes 1 and 2 explained 39.7 and 25.1% of the total variance in soil fungal community composition, respectively (Figure 5B). The fungal community compositions at the phylum level in the SMS-PE treatments were mainly related to a higher proportion of O-alkyl C. The fungal community compositions at the phylum level in the SMS-AB treatments were mainly related to higher contents of NO_3_^−^-N, HFOC, and HA-C, a higher AI value, and higher proportions of aromatic C and phenolic C in SOC.

**Figure 6:**
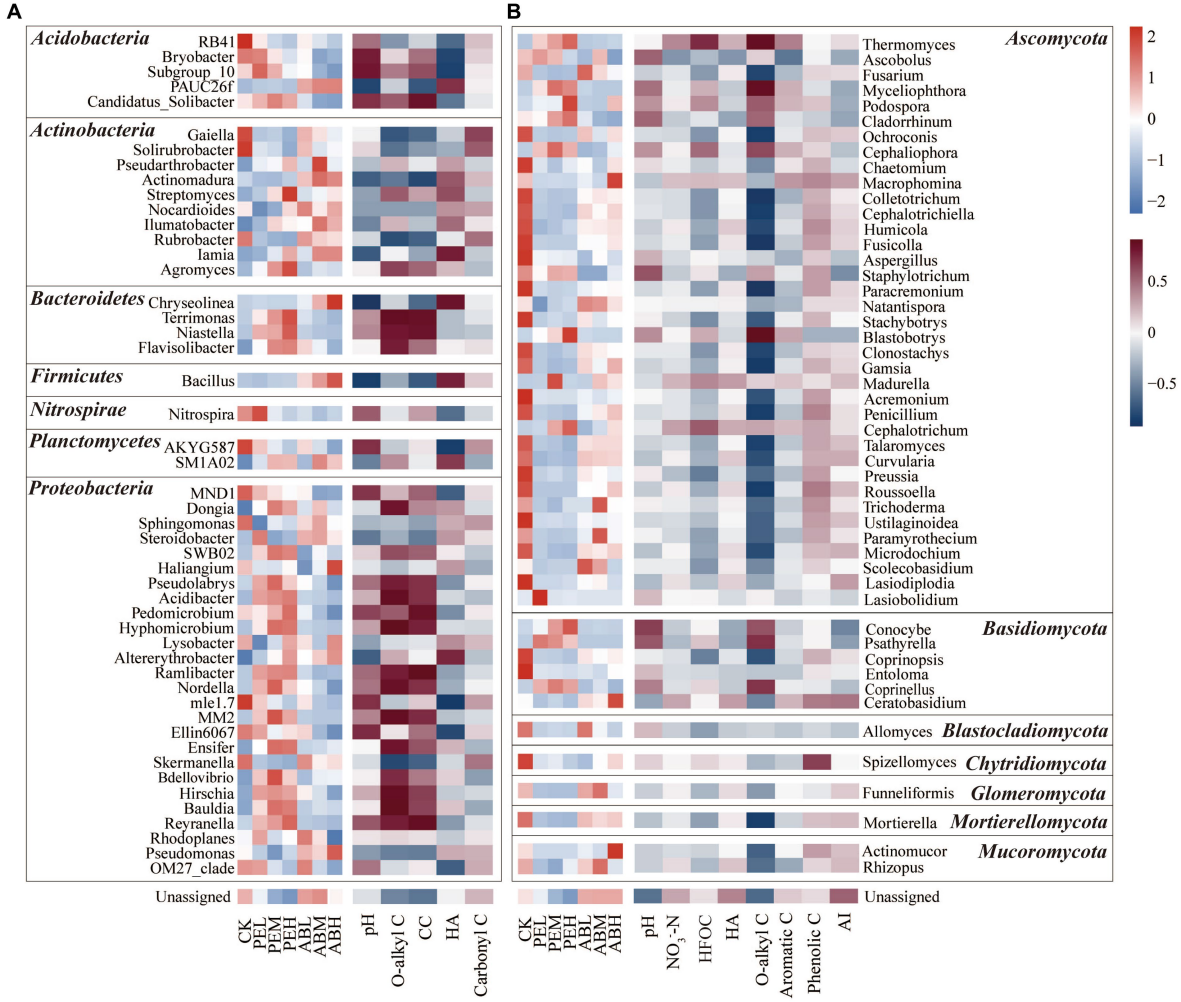
The top 50 most abundant bacterial **(A)** and fungal genera **(B)** in different treatments (the left sides of **A,B**) and their correlations with selected soil properties (the right sides of **A,B**). Both bacterial **(A)** and fungal **(B)** genera are grouped into seven phyla.

To further explore the influencing factors of bacterial and fungal community compositions at the genus level, Spearman correlation analysis was conducted between the top 50 most abundant genera and selected soil characteristics. SMS-PE application increased the relative abundances of *Acidibacter*, *Pedomicrobium*, *Hyphomicrobium*, *Ensifer*, *Hirschia*, *Bauldia*, *Reyranella*, *Terrimonas*, *Niastella*, *Flavisolibacter*, and *Agromyces*, which were positively correlated with soil O-alkyl C and CC proportions (Figure 6A). SMS-AB application increased the relative abundances of *PAUC26f*, *Actinomadura*, *Ilumatobacter*, *Chryseolinea*, and *Bacillus*, which were positively correlated with soil HA-C content. SMS-PE application markedly elevated the relative abundances of *Thermomyces*, *Myceliophthora*, *Cladorrhinum*, *Podospora*, *Cephaliophora*, *Blastobotrys*, *Conocybe*, and *Psathyrella*, which were positively correlated with O-alkyl C proportion in SOC (Figure 6B).

### 3.6 Prediction of potential functions of bacterial and fungal functional guild

**Figure 7:**
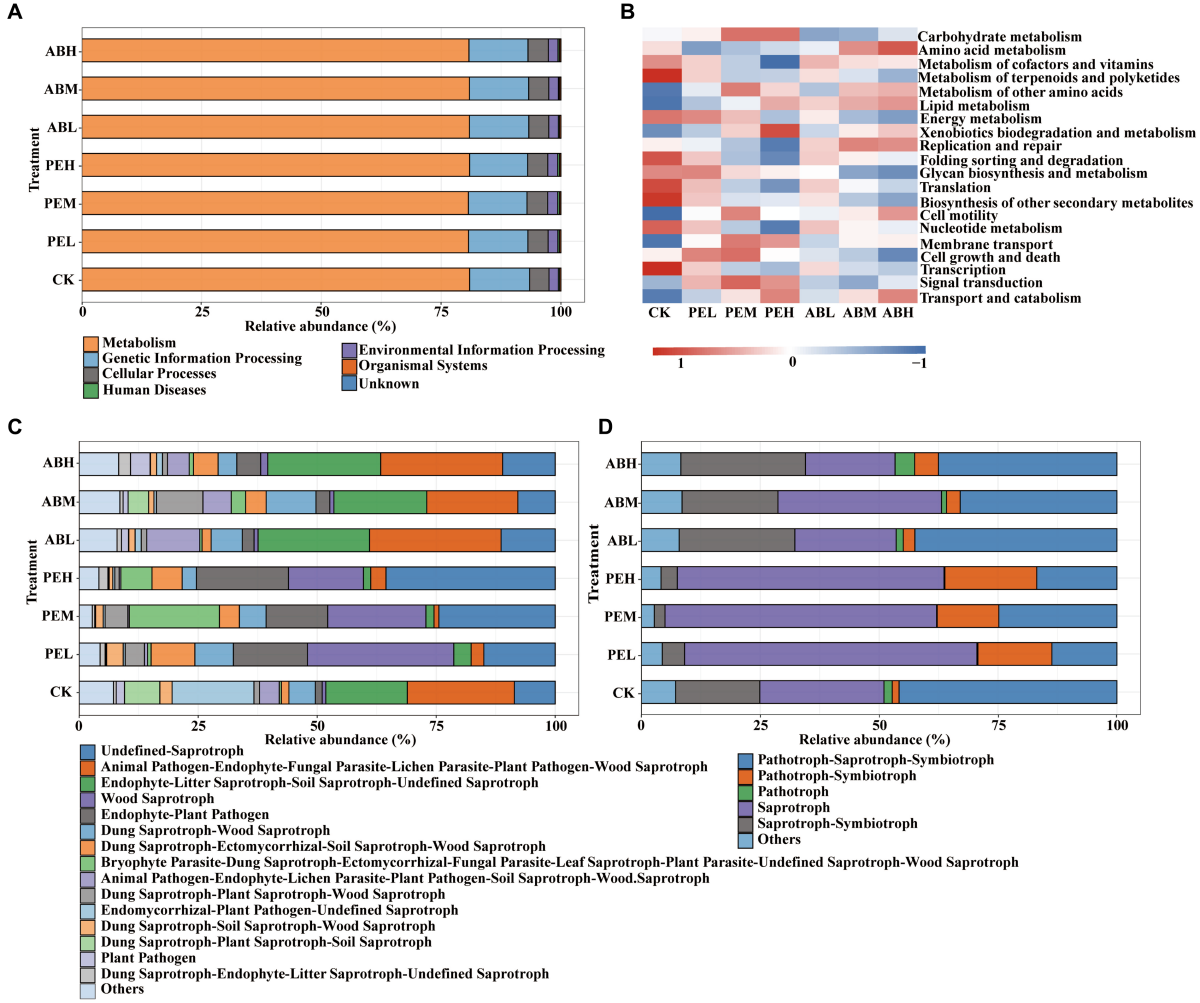
Relative abundances of KEGG L1 **(A)** and L2 **(B)** pathways predicted by PICRUSt2 for the soil bacterial communities and FUNGuild annotated fungal trophic modes **(C)** and main fungal functional guilds **(D)** in different treatments.

PICRUSt2 was used to predict the bacterial function of soil microorganisms based on KEGG pathways. The predicted pathways of bacteria in all treatments classified into seven functional categories (KEGG_L1): Metabolism (80.6–80.9%), Genetic Information Processing (12.1–13.6%), Cellular Processes (4.0–4.3%), Environmental Information Processing (1.9–2.0%), Human Diseases (0.22–0.36%), Organismal Systems (2.2–2.5%), and unknown (0.020–0.028%) (Figure 7A). Functions of soil bacteria predicted by PICRUSt2 indicated that the application of PE significantly increased soil carbohydrate metabolism (Figure 7B). The high-rate (8%) application of PE and AB significantly increased soil xenobiotic biodegradation and amino acid metabolism compared to other treatments, respectively.

Based on FUNGuild functional prediction, the functional classification information of fungi was obtained in different treatments, along with the abundance information of these functional classifications (Figure 7C). The prediction of fungi FUNGuild showed that the relative abundance of pathotroph-saprotroph-symbiotroph nutritional mode was higher than those of the other nutritional modes in both the CK and SMS-AB treatments, and it was significantly higher in CK (45.82%) than in the SMS-AB treatments (37.52–42.48%) (Figure 7D). By contrast, Saprotroph had a higher relative abundance (56.09–61.45%) than the other nutritional modes in the SMS-PE treatments.

## 4 Discussion

### 4.1 Variations in SOC component with different SMSs incorporation

Different SMSs incorporation significantly affected SOC component. Specifically, the application of SMS-PE increased active SOC components, including FA-C, LFOC, MBC, O-alkyl C, and CC, whereas SMS-AB mainly increased the stable components such as HFOC, HA-C, Aromatic C, thereby increasing the AI. Studies indicate that alterations in soil carbon components are closely linked to the input of exogenous organic matter (Hu et al., 2023). The substrate materials used for cultivating PE consisted of 20–35% sawdust, 5–25% corn cob, and 25–30% wheat straw (Yang F. et al., 2024; Yang G. et al., 2024). After the composting, the content of lignin, cellulose, and hemicellulose in the spent substrate of PE was determined to be 5.23, 18.04, and 8.05%, respectively (Supplementary Table S1). On the other hand, the substrates for cultivating AB contained 54% rice straw, 44.4% chicken manure, and other materials. The lignin, cellulose, and hemicellulose contents of the composted SMS-AB were much lower, 1.65, 4.42, and 2.15%, respectively (Supplementary Table S1). Wang et al. (2012) indicated that after the initial rapid decomposition phase of 6 months, wheat straw accumulates a higher amount of recalcitrant carbon compared to maize straw, which contains a more significant proportion of lignin and cellulose. During the decomposition process of wheat and maize straws (over 2 years), the contents of O-alkyl C and di-O-alkyl C decreased, whereas the stable carbon contents, e.g., alkyl C, aromatic C, phenolic C, and carbonyl C, gradually increased (Wang et al., 2012). The decreases in the contents of di-O-alkyl C and O-alkyl C in wheat and maize straws were associated with the decomposition of cellulose-like substances, whose decomposition products may be further metabolized and transformed into other compounds by microorganisms, increasing the contents of stable carbon components. Additionally, the humification index (HA/FA ratio) of SMS-AB was significantly higher than that of SMS-PE (Supplementary Table S1). Therefore, SMS-AB exhibited higher levels of recalcitrant C and AI. By contrast, SMS-PE had a higher proportion of CC (easily degradable carbon).

### 4.2 Soil microbial community composition and function under different SMSs incorporation

Involved in SOC turnover and nutrient transformation, soil microbes are crucial to ecosystem functions. This study demonstrates that the application of SMSs with varying carbon components significantly alters soil microbial community composition (Figure 3). The relative abundance of Proteobacteria was notably higher in SMS-PE, and its relative abundance positively correlated with the application rate of SMS. It could be attributed to the preference of Proteobacteria for decomposing labile organic carbon fractions, which were enriched by applying SMS-PE in the soil (Fierer et al., 2012). Comparing two SMSs, SMS-AB application enhanced the relative abundance of Firmicutes and Actinobacteria. Compared to the copiotrophic bacteria Proteobacteria, Firmicutes have a greater capacity for decomposing recalcitrant carbon sources (Ling et al., 2021). Actinobacteria, known for their versatile metabolism and rapid growth among soil bacteria, can promote soil carbon storage by producing polysaccharides, thereby enhancing the stability of soil carbon components (Mitra et al., 2022; Yang F. et al., 2024; Yang G. et al., 2024). This suggests that the application of AB led to an increase in the proportion of recalcitrant carbon in the soil compared to SMS-PE treatment. For fungi, comparing two SMSs, SMS-PE treatment increased the relative abundance of Ascomycota and Basidiomycota, whereas SMS-AB treatment led to a decrease. Ascomycota and Basidiomycota are primarily known for their saprophytic or parasitic nutritional modes, which could be attributed to the higher cellulose content in the SMS-PE treatment (Lundell et al., 2014). Fungi belonging to Ascomycota and Basidiomycota produce enzymes such as cellulases which are involved in cellulose degradation (Singh et al., 2003). Conversely, SMS-AB application increased the presence of soil’s recalcitrant C compounds.

The functional prediction results show that the SMS-PE application significantly increased the soil Carbohydrate metabolism (Figure 7B), involving the breakdown and metabolism of various organic compounds, including the degradation of hemi-cellulose and cellulose (Toledo et al., 2017). Bacterial functional predictions suggested that applying a high rate of PE (8%) significantly increased the relative abundance of xenobiotics biodegradation and metabolism in the soil. This may be attributed to the presence of microbial communities within the SMS-PE that exhibit the capability to degrade lignin compounds, which are universally recognized for their proficiency in decomposing dyes, hydrocarbons (notably polycyclic aromatic hydrocarbons), pesticides, and emerging contaminants (Leong et al., 2022a). Bacterial functional predictions indicated that the application of AB significantly increased the replication and repair function, which is beneficial for repairing pathogen infection (Wang et al., 2022). The high-rate application of AB (8%) significantly increased amino acid metabolism compared to other treatments. This may be due to the presence of poultry manure in AB, which can accelerate the metabolism of N-containing substances. The more abundant the bacteria with amino acid metabolism function, the more significant their promoting effect on amino acid production and humus synthesis (Wu et al., 2017).

The FUNGuild functional prediction reveals that Pathotroph-Saprotroph-Symbiotroph nutritional mode predominates in the CK and SMS-AB treatments, while the Saprotroph mode is most abundant in the PE treatment. It has been reported that saprotrophic fungi are the primary decomposers of dead or senescent plant material in soils, playing an essential role in the decomposition of organic matter and nutrient cycling (Duan et al., 2023). In contrast, pathogenic fungi obtain their nutrition from living host cells, negatively impacting crop growth (Anthony et al., 2017). The application of PE significantly reduced the relative abundance of Animal Pathogen-Endophyte-Lichen Parasite-Plant Pathogen-Soil Saprotroph-Wood Saprotroph, Animal Pathogen-Endophyte-Fungal Parasite-Lichen Parasite-Plant Pathogen-Wood Saprotroph, and Endophyte-Litter Saprotroph-Soil Saprotroph-Undefined Saprotroph, while increasing the relative abundance of “Wood Saprotroph and Endophyte-Plant Pathogen” (Figure 7C). The fungi corresponding to Animal Pathogen-Endophyte-Fungal Parasite-Lichen Parasite-Plant Pathogen-Wood Saprotroph are Nectriaceae, a family containing essential plant pathogens that induce disease in grapevines, avocados, and olives (Malapi-Wight et al., 2015). Within Nectriaceae, the most abundant genus is *Fusarium*. The fungi corresponding to Animal Pathogen-Endophyte-Lichen Parasite-Plant Pathogen-Soil Saprotroph-Wood Saprotroph are only *Fusarium*. *Fusarium* species are soil-borne vascular wilt pathogens and rank among the most crucial phytopathogenic and toxigenic fungi (Tang et al., 2020). Adding organic fertilizer likely promotes beneficial microbial growth while suppressing pathogenic bacteria in the soil (Hartmann et al., 2015). In this study, SMS-PE application might have increased beneficial fungi like *Cladorrhinum*, *Podospora*, and *Blastobotrys*, inhibiting the growth of harmful fungi such as *Fusarium* (McCormick et al., 2012; Zhao et al., 2021). In this study, SMS-PE application might have increased beneficial fungi like *Cladorrhinum*, *Podospora*, and *Blastobotrys*, inhibiting the growth of harmful fungi such as *Fusarium* (McCormick et al., 2012; Zhao et al., 2021). Wang et al. (2020) has shown that SMS application can inhibit soil pathogens, alter the rhizosphere microbial community of cucumbers, and enhance cucumber growth. These findings collectively suggest that SMS can be developed into a biocontrol-functional organic fertilizer. Moreover, bioactive compounds with biocontrol properties can be extracted from SMS using appropriate methods and incorporating them into liquid fertilizers can create effective liquid biocontrol fertilizers (Qin et al., 2023).

### 4.3 The relationship between SOC component and microbial community composition

The aim of this study was to investigate the relationship between soil microbial community structure and SOC composition in soils applied with different types of SMSs. The mantel test showed that the phylum-level bacterial community composition was significantly affected by soil pH, O-alkyl C, CC, HA-C, and carbonyl C, whereas the phylum-level fungal community composition was significantly affected by soil pH, NO_3_^−^, HFOC, HA-C, O-alkyl C, aromatic C, phenolic C, and AI (Figure 4). The PCA analysis showed that the phylum-level bacterial community compositions in the SMS-PE treatments were mainly associated with the relatively high proportions of O-alkyl C and CC. The O-alkyl C and CC were positively correlated with the relative abundance of Proteobacteria, aligning with the finding of Di Lonardo et al. (2017) that Proteobacteria abundance was significantly correlated with easily degradable compounds. Further corroboration comes from Bonanomi et al. (2019), who noted that decreases in the proportions of O-alkyl C and di-O-alkyl C during leaf litter decomposition contribute to the reduction in the relative abundance of Proteobacteria. The bacterial community compositions at the phylum level in the SMS-AB treatments were mainly related to the relatively high content of HA-C. HA-C was positively correlated with the relative abundances of Firmicutes and Actinobacteria, which was similar to the finding of Li et al. (2019) that the application of HA increased the relative abundances of Firmicutes and Actinobacteria in soil. Actinobacteria are vital players in organic matter transformation and play a significant role in breaking down persistent polymers (Xu et al., 2016). The fungal community compositions at the phylum level in the SMS-PE treatments were mainly related to the relatively high proportion of O-alkyl C. O-alkyl C was positively correlated with the relative abundances of Basidiomycota and Ascomycota. This suggests that Ascomycota and Basidiomycota may produce more O-alkyl C, and thus, more organic matter is transformed by these microorganisms.

As aforementioned, the application of SMS-PE mainly increased the relative abundance of Proteobacteria. Specifically, the Proteobacteria genera of *Acidibacter*, *Pedomicrobium*, *Hyphomicrobium*, *Ensifer*, *Hirschia*, *Bauldia*, and *Reyranella* increased in relative abundance with a higher application rate of SMS-PE. In addition, the application of SMS-PE also increased the relative abundances of *Terrimonas*, *Niastella*, and *Flavisolibacter* in Bacteroidetes and *Agromyces* in Actinobacteria. The relative abundances of these genera showed positive correlations with O-alkyl C and CC. *Hyphomicrobium*, *Pedomicrobium*, *Reyranella*, and *Bauldia* are the major denitrifying bacteria in soil, with the ability to reduce nitrate to nitrite and further to NO and N_2_O (Seitzinger et al., 2006). *Ensifer* and *Niastella* were reported to be associated with NO_3_^−^-N and COD, respectively, suggesting that they may be involved in denitrification processes (Guo et al., 2023). The increased labile C (O-alkyl C and CC) in the SMS-PE treatments can act as electron donors for the denitrification processes (Cui et al., 2023), which explains the observed increased relative abundances of the bacteria associated with denitrification. *Hirschia* and *Terrimonas* can secrete cellulases to degrade lignocellulosic fibers (Martin et al., 2014). *Agromyces* strains are known to produce β-glucosidase during the decomposition of rice straw (Han et al., 2023). *Flavisolibacter* can degrade cellulose and is considered a beneficial bacterium that plays a key role in improving plant disease resistance, promoting plant growth, and fixing carbon dioxide (Liu et al., 2016). The application of SMS-AB led to increases in the relative abundances of *PAUC26f* in Acidobacteria, *Actinomadura* and *Ilumatobacter* in Actinobacteria, *Chryseolinea* in Bacteroidetes, and *Bacillus* in Firmicutes. In addition, the relative abundances of these genera were positively correlated with the content of HA-C. *Actinomadura* and *Ilumatobacter* are involved in the biosynthesis of antibiotics such as polyketones, streptomycin, and vancomycin (Hertweck, 2009). Additionally, HA application promotes the growth of *Ilumatobacter* (Sang et al., 2021). *Chryseolinea* can degrade complex organic compounds such as polysaccharides (Milkereit et al., 2021). *Bacillus* is recognized as an efficacious biocontrol agent, mitigating the prevalence of soil-borne diseases, and improving the soil ecosystem (Liang et al., 2018). A study showed that adding 0.5% *Bacillus subtilis* to cow manure compost promoted the conversion of organic C to humus (Duan et al., 2020). HA is an organic macromolecule produced by microbial degradation of plant, animal, and microbial residues (Yu et al., 2019). Mainly composed of aromatic compounds rich in active functional groups (phenolic and carboxyl groups), HA is difficult to mineralize. Inoculating microbes to improve the composting efficiency of SMS and elevate the levels of functional substances, such as humic acids, provides a new strategy for creating functional fertilizers derived from SMS (Sun et al., 2023; Li et al., 2024).

The application of SMS-PE significantly increased the relative abundances of *Thermomyces*, *Myceliophthora*, *Cladorrhinum*, *Podospora*, *Cephaliophora*, and *Blastobotrys* in Ascomycota and *Conocybe* and *Psathyrella* in Basidiomycota. The relative abundances of these genera were positively correlated with the relative abundance of O-alkyl C (Figure 6B). There was un-degraded cellulose in the composted SMS-PE, and *Myceliophthora* and *Thermomyces* can secrete abundant cellulases and xylanases to degrade the cellulose (Van den Brink et al., 2012). *Cladorrhinum*, *Podospora*, and *Blastobotrys* are potential biocontrol agents of plant pathogens and promote plant growth. In addition, they produce enzymes with biotechnological applications (Barrera et al., 2019). *Podospora* and *Blastobotrys*, abundant in healthy soils, can reduce tomato wilt disease (*Verticillium*) (Dutta, 1981). *Cephaliophora*, a predatory fungus, is under-researched (Morikawa et al., 1993). *Psathyrella*, a wood-decomposer (Suetsugu et al., 2020) whose secondary metabolites have antibacterial and inhibitory properties, is a potential biocontrol agent. *Conocybe* is capable of cellulose degradation, polysaccharide production, and pathogenic fungi inhibition (Yang et al., 2022). Investigating the interactions between SOC components and distinct microbial genera, as well as their functional roles, establishes a basis for future development of SMS-derived functional fertilizers. While the SMS-PE application significantly altered the abundances of various fungal genera, suggesting potential benefits for soil health and biocontrol, these laboratory-based findings must be validated through field experiment. Additionally, future research should consider the initial microbial community composition and physical soil properties, which can significantly influence SOC fractions and microbial community responses.

## 5 Conclusion

Our research indicates a close relationship between SOC composition and microbial community and function under the short-term application of SMS. The application of SMS-PE resulted in higher proportions of CC and O-alkyl C, which stimulated the bacterial and fungal genera associated with cellulose degradation. In addition, functional predictions indicated that the relative abundance of carbohydrate metabolism in bacteria was significantly increased, and the relative abundances of fungal pathogens, especially *Fusarium* spp., were notably decreased. The application of SMS-AB increased AI, the proportion of aromatic C, and the contents of HA-C and HFOC. The relative abundances of beneficial genera like *Actinomadura*, *Ilumatobacter*, and *Bacillus* were increased. Furthermore, functional prediction revealed that high-rate SMS-AB application elevated bacterial amino acid metabolism and the abundance of plant pathogenic fungi. Our results elucidate that the application of different SMSs can alter SOC composition, thereby affecting the composition and function of soil microbial communities. This work enhances our understanding of the relationship between soil organic carbon components and microbial communities and provides foundational insights for developing effective SMS management strategies.

## Data availability statement

The datasets presented in this study can be found in online repositories. The names of the repository/repositories and accession number(s) can be found at: NCBI - PRJNA 1051179 and PRJNA 1051023.

## Author contributions

GY: Conceptualization, Data curation, Validation, Visualization, Writing – original draft, Writing – review & editing. YM: Funding acquisition, Supervision, Writing – review & editing. XM: Formal analysis, Methodology, Visualization, Writing – review & editing. XW: Resources, Software, Validation, Writing – review & editing. CL: Resources, Supervision, Writing – review & editing. WX: Formal analysis, Visualization, Writing – review & editing. JL: Project administration, Writing – review & editing. DG: Project administration, Resources, Writing – review & editing.

## Footnotes

^1^ http://www.funguild.org/
