# Supplementary material for: Changes in soil organic carbon components and microbial community following spent mushroom substrate application
Source: Front Microbiol. 2024 May 17;15:1351921. doi: 10.3389/fmicb.2024.1351921 (PMC11140037; doi:10.3389/fmicb.2024.1351921)
Supplement: Supplementary file 1 [file Table_1.DOCX]

**Changes in Soil Organic Carbon Components and Microbial Community Following Spent Mushroom Substrate Application**

Guiting Yang^1^, Yan Ma^123*^, Xiaochi Ma^1^, Xuanqing Wang^1^, Chao Lu^1^, Wenyi Xu^14^, Jia Luo^1^, Dejie Guo^1^

*^1^* *Institute of Agricultural Resources and Environment, Jiangsu Academy of Agricultural Sciences, Nanjing 210014, China*.

*^2^ Key Laboratory of Saline-Alkali Soil Improvement and Utilization (Coastal Saline-Alkali Lands), Ministry of Agriculture and Rural Affairs, Nanjing 210014, China*.

*^3^* *National Agricultural Experiment Station for Agricultural Environment, Luhe, Nanjing 211500, China*.

*^4^ Department of Geosciences and Natural Resource Management, University of Copenhagen, 1350 Copenhagen K, Denmark*.

Correspondence Yan Ma, E-mail: [myjaas@sina.com](mailto:myjaas@sina.com), Phone: 86-25-8439-0248.

Table S1. Basic properties of different types of SMSs.

| Properties | SMS-PE | SMS-AB |
| --- | --- | --- |
| C (%) | 34.23±0.98^a^ | 19.70±1.47^b^ |
| N (%) | 2.67±0.18^a^ | 1.96±0.07^b^ |
| P (%) | 1.03±0.05^a^ | 0.72±0.09^b^ |
| K (%) | 0.93±0.06^a^ | 1.97±0.09^b^ |
| DOC (%) | 6.10±0.09^a^ | 1.02±0.02^b^ |
| Lignin (%) | 5.23±0.25^a^ | 1.65±0.17^b^ |
| Cellulose (%) | 18.04±1.03^a^ | 4.42±0.33^b^ |
| Hemicellulose (%) | 8.05±0.14^a^ | 2.15±0.23^b^ |
| FA-C (%) | 8.93±0.62^a^ | 2.80±0.09^b^ |
| HA-C (%) | 9.94±0.29^a^ | 9.14±0.44^a^ |
| HA/FA | 1.11±0.10^b^ | 3.26±0.27^a^ |

DOC, Dissolved organic C.

Table S2. Soil properties under different treatments after 115 days of incubation.

| Treatments | pH | SOC  (g kg^-1^) | DOC  (g kg^-1^) | MBC  (g kg^-1^) | AP  (mg kg^-1^) | TN  (g kg^-1^) | TP  (g kg^-1^) | NO_3_^-^-N  (mg kg^-1^) | NH_4_^+^-N  (mg kg^-1^) | |
| --- | --- | --- | --- | --- | --- | --- | --- | --- | --- | --- |
| CK | 8.53a | 24.77e | 128.6e | 329.8f | 9.68f | 1.48e | 0.40e | 119.6f | | 11.53c |
| PEL | 8.33b | 35.93c | 357.6c | 580.7cd | 25.96d | 2.79c | 0.48c | 158.2e | | 12.78ab |
| PEM | 8.31b | 42.66b | 568.3b | 802.5b | 44.10b | 3.69b | 0.57b | 259.6d | | 12.99a |
| PEH | 8.14c | 51.98a | 789.7a | 1076a | 64.46a | 4.78a | 0.70a | 296.4c | | 13.26a |
| ABL | 7.94d | 29.23d | 266.5d | 443.8e | 19.35e | 2.03d | 0.44d | 255.3d | | 12.03bc |
| ABM | 7.74e | 33.10cd | 366.4c | 590.2c | 27.71d | 2.53c | 0.48c | 366.2b | | 11.36c |
| ABH | 7.60f | 42.50b | 554.7b | 518.1d | 35.66c | 3.54b | 0.56b | 447.3a | | 11.44c |
| Types of SMS (T) | a | a | a | a | a | a | a | a | | a |
| Application rate of SMS (A) | a | a | a | a | a | a | a | a | | a |
| T×A | a | ns | a | a | a | ns | a | ns | | a |

Values are mean (n = 4). Different letters indicate significant differences between different treatments at *P* < 0.05. SOC, soil organic C; DOC, dissolved organic C; MBC, [soil microbial biomass](https://www.sciencedirect.com/topics/earth-and-planetary-sciences/microbial-biomass-in-soil) C; AP, available P; TN, total N; TP, total P. ^a^ Indicate the significant differences at *P* < 0.05; ns, no significance.

Table S3.  Soil organic C fractions under different treatments after 115 days of incubation.

| Treatments | LFOC (g kg^-1^) | HFOC (g kg^-1^) | FA-C (g kg^-1^) | HA-C (g kg^-1^) |
| --- | --- | --- | --- | --- |
| CK | 3.51e | 20.25d | 1.89e | 6.31f |
| PEL | 5.54c | 28.68bc | 2.90cd | 7.00e |
| PEM | 11.94b | 28.44bc | 3.67b | 8.99d |
| PEH | 17.68a | 30.66b | 4.86a | 10.26c |
| ABL | 4.03de | 24.54c | 2.62d | 9.18d |
| ABM | 4.79cd | 27.72bc | 3.06c | 11.30b |
| ABH | 4.98cd | 35.36a | 3.59b | 12.90a |
| Types of SMS (T) | a | ns | a | a |
| Application amount of SMS (A) | a | a | a | a |
| T×A | a | a | a | ns |

Values are mean (n = 4). Different letters indicate significant differences between different treatments at *P* < 0.05. LFOC, light fraction organic C (LFOC); HFOC, heavy fraction organic carbon; TN, total N; TP, total P; FA-C, fulvic acid; HA-C, humic acid. ^a^ Indicate the significant differences at *P* < 0.05, ns, no significance.

**Text S1: Soil Property Analysis**

The soil pH was measured using a pH electrode with a soil-to-water ratio 1:2.5 (w/v). Mineral N (NH_4_^+^ and NO_3_^-^) in the soil was extracted using a 0.05 M CaCl_2_ solution and quantified using an AA3 continuous flow autoanalyzer (Bran & Luebbe, Germany) (Wei et al., 2017). Soil AP was extracted using 0.5 M NaHCO_3_ and measured at 700 nm using UV spectrophotometry (Olsen, 1982). Soil TN was determined using a dry combustion method with an elemental analyzer (Elementar, Germany).

**Reference**

Wei, X., Hu, Y., Peng, P., Zhu, Z., Atere, C. T., O’donnell, A. G., Wu, J., Ge, T. (2017). Effect of P stoichiometry on the abundance of nitrogen-cycle genes in phosphorus-limited paddy soil. Biology and Fertility of Soils, 53, 767-776.

Olsen, S. (1982). Anion resin extractable phosphorus. Methods of Soil Analysis, 2, 423-424.
